# Spt5 modulates cotranscriptional spliceosome assembly in *Saccharomyces cerevisiae*

**DOI:** 10.1261/rna.070425.119

**Published:** 2019-10

**Authors:** Isabella E. Maudlin, Jean D. Beggs

**Affiliations:** Wellcome Centre for Cell Biology, School of Biological Sciences, University of Edinburgh, Edinburgh EH9 3BF, United Kingdom

**Keywords:** transcription, pre-mRNA splicing, yeast

## Abstract

There is increasing evidence from yeast to humans that pre-mRNA splicing occurs mainly cotranscriptionally, such that splicing and transcription are functionally coupled. Currently, there is little insight into the contribution of the core transcription elongation machinery to cotranscriptional spliceosome assembly and pre-mRNA splicing. Spt5 is a member of the core transcription elongation machinery and an essential protein, whose absence in budding yeast causes defects in pre-mRNA splicing. To determine how Spt5 affects pre-mRNA splicing, we used the auxin-inducible degron system to conditionally deplete Spt5 in *Saccharomyces cerevisiae* and assayed effects on cotranscriptional spliceosome assembly and splicing. We show that Spt5 is needed for efficient splicing and for the accumulation of U5 snRNPs at intron-containing genes, and therefore for stable cotranscriptional assembly of spliceosomes. The defect in cotranscriptional spliceosome assembly can explain the relatively mild splicing defect as being a consequence of the failure of cotranscriptional splicing. Coimmunoprecipitation of Spt5 with core spliceosomal proteins and all spliceosomal snRNAs suggests a model whereby Spt5 promotes cotranscriptional pre-mRNA splicing by stabilizing the association of U5 snRNP with spliceosome complexes as they assemble on the nascent transcript. If this phenomenon is conserved in higher eukaryotes, it has the potential to be important for cotranscriptional regulation of alternative splicing.

## INTRODUCTION

Genes in most eukaryotes contain noncoding sequences (“introns”) that interrupt the coding sequences (“exons”). Introns are present in the nascent transcripts (pre-mRNAs) and are excised and the exons joined in a process called pre-mRNA splicing. Introns are defined by short conserved sequences: the 5′ splice site (5′SS), the 3′SS, and the branch point (BP). *Trans*-acting factors recognize these motifs and position the pre-mRNA for the two transesterification reactions catalyzed by the spliceosome. The spliceosome is a large macromolecular complex composed of small nuclear ribonucleoprotein particles (snRNPs)—U1, U2, U4/U6, and U5—and many non-snRNP proteins (for review, see Hoskins and Moore 2012). Both in vitro and in vivo, the snRNPs assemble on the pre-mRNA in a stepwise manner. First, the U1 snRNP binds to the 5′SS, and the U2 snRNP binds to the BP, forming the prespliceosome, or A complex. The U4/U6•U5 tri-snRNP then joins, forming the pre-B intermediate complex, which is unstable (Boesler et al. 2016). The pre-B complex undergoes substantial rearrangements to produce the B complex in which the tri-snRNP is stably associated. The spliceosome undergoes further structural rearrangements to form the catalytically active B* complex, which catalyzes the first step of splicing. Further rearrangements promote the second catalytic step that generates the spliced RNA and then the spliceosome dissociates. The splicing factors are then recycled for a new round of splicing (for review, see Will and Lührmann 2011).

There is increasing evidence from lower to higher eukaryotic organisms that splicing occurs mainly cotranscriptionally—that is, spliceosomes assemble and splicing catalysis occurs as RNA polymerase II (RNAPII) transcribes along the gene, before transcription termination (Kotovic et al. 2003; Görnemann et al. 2005; Lacadie and Rosbash 2005; Listerman et al. 2006; Carrillo Oesterreich et al. 2010, 2016; Ameur et al. 2011; Khodor et al. 2012; Tilgner et al. 2012; Brugiolo et al. 2013; Nojima et al. 2015; Harlen et al. 2016; Wallace and Beggs 2017). By definition, cotranscriptional splicing occurs in close proximity to the transcription elongation machinery, and it is well-established that transcription and splicing are functionally coupled such that they influence one another (Fong and Zhou 2001; de la Mata et al. 2003; Howe et al. 2003; Alexander et al. 2010a; Ip et al. 2011; Braberg et al. 2013; Chathoth et al. 2014; Dujardin et al. 2014; Fong et al. 2014; Aslanzadeh et al. 2018). There are two nonmutually exclusive models for how transcription affects splicing: (i) the speed of RNAPII elongation affects intron/exon recognition (termed the “kinetic” model); and/or (ii) the transcription elongation machinery facilitates recruitment of splicing factors to the site of transcription (termed the “recruitment” model) (for review, see Kornblihtt et al. 2004; Bentley 2005, 2014; Perales and Bentley 2009; de la Mata et al. 2011; Dujardin et al. 2013; Merkhofer et al. 2014).

Spt5 is the most highly conserved core transcription elongation factor that, following initiation of transcription, associates tightly with RNAPII during elongation until transcription termination, and acts as a docking site for protein complexes that influence RNAPII processivity, RNA processing, and histone modifications (for review, see Hartzog and Fu 2013). It is thought that Spt5 enhances RNAPII processivity by stabilizing the interaction between its clamp domain and the DNA template (Hirtreiter et al. 2010; Klein et al. 2011; Martinez-Rucobo et al. 2011). In metazoans, DSIF (Spt4/5 in *Saccharomyces cerevisiae*) and NELF cause RNAPII to pause in a stable manner downstream from the transcription start sites, referred to as promoter-proximal pausing (for review, see Adelman and Lis 2012). Depletion of Spt5 in *Schizosaccharomyces pombe* causes genome-wide defects in transcription elongation (Shetty et al. 2017). In mammals, Spt5 depletion does not cause such genome-wide defects but seems to be important for elongation only on long genes (Fitz et al. 2018). Spt5 has a conserved but nonessential carboxy-terminal region (CTR) that is differentially phosphorylated during the course of transcription, and is important for RNAPII elongation and histone modification (Zhou et al. 2009). In particular, phosphorylation of the CTR of Spt5 by the Bur1/2 kinase complex is important for Paf1 complex (Paf1C) recruitment to elongating RNAPII (Laribee et al. 2005; Liu et al. 2009). Paf1C is associated with RNAPII along actively transcribed genes where it serves as a “platform” that coordinates the association of transcription factors and chromatin-modifying enzymes with RNAPII, thereby facilitating transcription elongation (for review, see Jaehning 2010). Paf1C is required for H2BK123 monoubiquitination, which in turn is required for H3K4 di- and trimethylation (Krogan et al. 2003; Ng et al. 2003; Wood et al. 2003; Xiao et al. 2005b). The Paf1 complex also affects H3K36 trimethylation (Chu et al. 2007).

There is evidence that Spt5 affects the pre-mRNA splicing outcome. For example, mutations in Spt5 or its partner, Spt4, result in splicing defects in *S. cerevisiae* (Lindstrom et al. 2003; Burckin et al. 2005; Xiao et al. 2005a), and depletion of Spt4 in mammalian cells results in changes to alternative splicing patterns (Liu et al. 2012). Further, depletion of Spt5 in mammalian cells causes pre-mRNA accumulation on some genes (Diamant et al. 2012). Similarly, depletion of Spt5 in *S. pombe* causes pre-mRNA accumulation, as shown by RNA sequencing (Shetty et al. 2017). Additionally, it was shown in yeast that Spt5 is enriched on intron-containing genes compared to intronless genes (known as “intron bias”) and that Spt5 coimmunoprecipitates with Prp40, a core protein of the U1 snRNP (Moore et al. 2006). Further, Spt5 was found to crosslink more to pre-mRNA intron sequences compared to exon sequences in *S. cerevisiae* (Battaglia et al. 2017).

Collectively, these studies demonstrate that Spt5 is important for splicing outcome, but there is no clear insight into how this happens. As Spt5 functions during transcription, it seems likely that it affects splicing cotranscriptionally although, apparently, this has not been investigated. Here, an auxin-inducible degron (AID) system (Nishimura et al. 2009; Mendoza-Ochoa et al. 2018) was used to conditionally deplete Spt5 in *S. cerevisiae*, and effects on cotranscriptional spliceosome assembly and splicing were investigated. Analysis of cotranscriptional spliceosome assembly showed that depletion of Spt5 did not affect cotranscriptional U1 or U2 snRNP recruitment, meaning at least the prespliceosome or A complex can form cotranscriptionally in the absence of Spt5. However, cotranscriptional recruitment of the U5 snRNP was reduced, indicating that B complex cannot efficiently or stably form cotranscriptionally in the absence of Spt5. Further, Spt5 pulls down all spliceosomal snRNAs and coimmunoprecipitates with spliceosomal proteins. We propose that Spt5 affects U5 snRNP recruitment and pre-B and/or B complex formation cotranscriptionally through interaction with components of the spliceosome. Together, these data provide insight into how Spt5 could specifically affect cotranscriptional pre-mRNA splicing to cause a mild splicing defect in *S. cerevisiae*.

## RESULTS

### Use of the AID system to conditionally deplete Spt5

**FIGURE 1.**
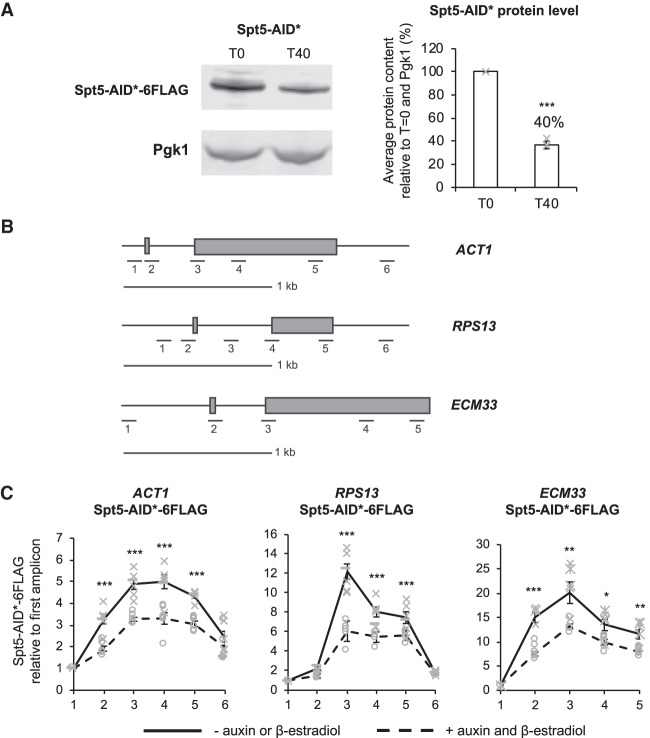
Use of the AID system to conditionally deplete Spt5. (*A*) Western blot probed with anti-Flag and anti-Pgk1 as a loading control. Samples were taken before (T0) and 40 min (T40) after addition of auxin and β-estradiol. Spt5-AID* depletion was quantified and shown as the percentage mean of three biological replicates for T40 relative to T0 and normalized to the Pgk1 signal. Error bars, standard error of the mean. Gray crosses indicate the individual replicate values. (*B*) A diagram is drawn to scale, showing the positions of amplicons used for ChIP-qPCR analyses across each of the intron-containing genes *ACT1*, *RPS13*, *ECM33.* Exons are represented by gray rectangles and a scale bar of 1 kb is shown. (*C*) Anti-Flag ChIP followed by qPCR analysis of the intron-containing genes *ACT1*, *RPS13*, *ECM33* without (−) auxin and β-estradiol (solid black line) or (+) 40 min after auxin and β-estradiol (dashed black line) addition to depleting Spt5-AID*-6Flag. The *x*-axis of each graph shows the amplicons used for ChIP-qPCR analysis. The data are presented as the mean percentage of input relative to the first amplicon of each gene for at least three biological replicates. Error bars, standard error of the mean. Asterisks show the statistical significance (Student's unpaired *t*-test). (*) *P* < 0.05, (**) *P* < 0.01, and (***) *P* < 0.001. Not significant, *P* > 0.05. Gray crosses indicate the individual replicate values without auxin and β-estradiol, and gray circles indicate the individual replicate values 40 min after auxin and β-estradiol addition.

To determine whether the physical presence of Spt5 affects cotranscriptional spliceosome assembly in vivo in *S. cerevisiae*, Spt5 was conditionally depleted using the AID system. Spt5 was carboxy-terminally tagged with the AID* degron and 6xFlag epitope in a strain that allowed conditional induction with β-estradiol of OsTIR1, the auxin-binding receptor protein from *Oryza sativa* (McIsaac et al. 2014; Mendoza-Ochoa et al. 2018). Following the addition of β-estradiol and auxin to the culture, the auxin-bound OsTIR1 targets the Spt5-AID* protein for ubiquitylation and degradation by the proteasome. Western blotting showed that treatment for 40 min resulted in the reduction of Spt5-AID* to 40%, on average, of the undepleted amount (Fig. 1A). Chromatin immunoprecipitation and quantitative PCR (ChIP-qPCR) analysis across three intron-containing genes (Fig. 1B) showed that, in wild-type conditions, Spt5-AID* occupancy peaks over introns and exon 2 of the genes analyzed (Fig. 1C). After auxin treatment, Spt5-AID* was significantly depleted at each of the intron-containing genes tested (Fig. 1C).

### Depletion of Spt5 reduces the cotranscriptional recruitment of the U5 snRNP without affecting cotranscriptional prespliceosome assembly

**FIGURE 2.**
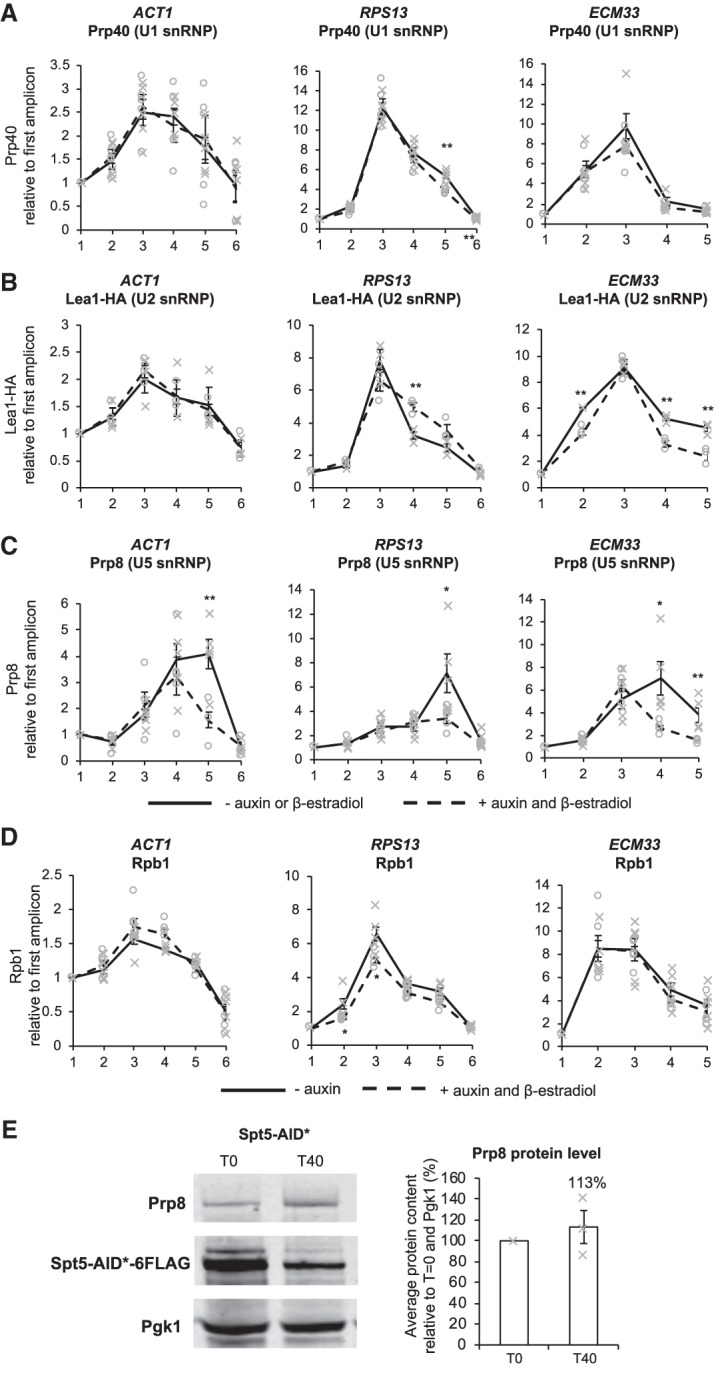
Depletion of Spt5 reduces cotranscriptional recruitment of U5 snRNPs. (*A*) Anti-Prp40 (U1 snRNP), (*B*) anti-Lea1-HA (U2 snRNP), (*C*) anti-Prp8 (U5 snRNP), and (*D*) anti-Rpb1 (RNAPII) ChIP and qPCR across intron-containing genes *ACT1*, *RPS13*, and *ECM33* without auxin or β-estradiol (solid black line) and with 40 min of auxin and β-estradiol treatment to deplete Spt5-AID* (dashed black line). The *x*-axes show the amplicons used for ChIP-qPCR analysis (see Fig. 1B). The ChIP data are presented as the mean percentage of input relative to the first amplicon of each gene for at least three biological replicates. Error bars, standard error of the mean. Gray crosses indicate the individual replicate values without auxin and β-estradiol and gray circles indicate the individual replicate values 40 min after auxin and β-estradiol addition. (*E*) Western blot probed with anti-Prp8 (U5 snRNP), anti-Flag, and anti-PGK1 as a loading control. T0, samples taken before, and T40, 40 min after addition of auxin and β-estradiol. Quantification of Prp8 is presented as the percentage mean of three biological replicates for T40 samples relative to T0 values and normalized to the PGK1 signal. Error bars, standard error of the mean. Asterisks show the statistical significance (Student's unpaired *t*-test). (*) *P* < 0.05, (**) *P* < 0.01, and (***) *P* < 0.001. Not significant, *P* > 0.05. Gray crosses indicate the individual replicate values.

As splicing factors assemble cotranscriptionally, their close proximity to chromatin enables them to be cross-linked to the DNA template and analyzed by ChIP-qPCR. In this way, the cotranscriptional recruitment of splicing factors and spliceosome assembly can be monitored in vivo (Kotovic et al. 2003; Görnemann et al. 2005; Lacadie and Rosbash 2005; Tardiff and Rosbash 2006). ChIP was performed, using antibodies against core members of the spliceosome, to determine whether depletion of Spt5 affects cotranscriptional spliceosome assembly at the intron-containing genes *ACT1*, *RPS13*, and *ECM33*. These three genes are well expressed and their transcripts are cotranscriptionally spliced (Wallace and Beggs 2017). Antibodies were used that detect Prp40 (U1 snRNP), Lea1-3HA (U2 snRNP) or Prp8 (U5 snRNP), which allowed a determination of which stage, if any, of cotranscriptional spliceosome assembly may be affected by depletion of Spt5. In conditions without auxin or β-estradiol, the ChIP profiles of U1 snRNP (Prp40), U2 snRNP (Lea1-3HA), and U5 snRNP (Prp8), were as expected; the U1 and U2 snRNP signals peaked near the 3′SS, and the U5 snRNP peaked nearer the 3′end of the gene. ChIP-qPCR showed that depletion of Spt5 for 40 min did not significantly or consistently affect U1 or U2 snRNP occupancies on the intron-containing genes tested (Fig. 2A,B), relative to conditions without depletion. In contrast, depletion of Spt5 resulted in a significant reduction in U5 snRNP occupancy where it normally peaks on *ACT1* (amplicon 5, exon 2)*,* on *RPS13* (amplicon 5, exon 2) and on *ECM33* (amplicons 4 and 5, exon 2) (Fig. 2C). Moreover, the U5 ChIP signal declined prematurely compared with normal.

It is conceivable that reduced U5 snRNP recruitment could be an indirect consequence of reduced RNAPII occupancy following Spt5 depletion, for example, causing loss of interactions between certain splicing factors and RNAPII. However, ChIP using an antibody against RNAPII (Rpb1) (Fig. 2D) showed no consistent effect on RNAPII occupancy across these intron-containing genes. Moreover, western blotting, performed with extracts from cells grown with or without 40 min of auxin treatment, showed no significant difference in the total cellular level of Prp8 protein upon Spt5 depletion (Fig. 2E), indicating that the observed loss of U5 snRNP occupancy, as measured by ChIP of Prp8 following Spt5 depletion, was not simply due to a reduction in the total cellular level of the Prp8 protein.

### Depletion of Spt5 causes defects in pre-mRNA splicing

**FIGURE 3.**
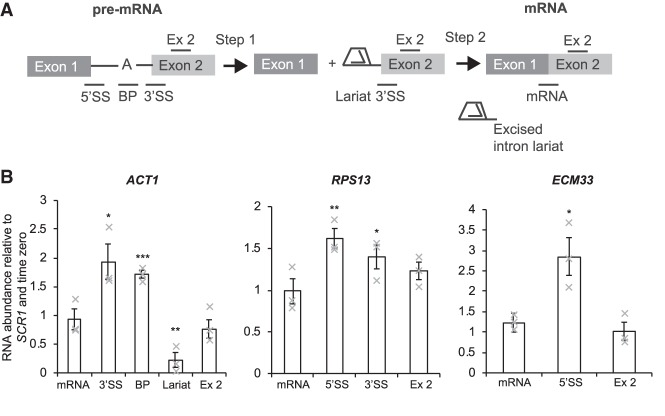
Depletion of Spt5 affects pre-mRNA splicing. (*A*) Cartoon showing the RT-qPCR amplicons for splicing analysis for an average gene. These detect pre-mRNA (5′SS or BP and 3′SS), lariat (excised intron or intron–exon 2), exon 2 (ex 2), and mRNA. (*B*) RT-qPCR analysis of the intron-containing genes *ACT1*, *RPS13*, and *ECM33* after depletion of Spt5-AID*, normalized to the *SCR1* RNAPIII transcript and time zero (without auxin and β-estradiol addition). Mean of three biological replicates. Error bars, standard error of the mean. Asterisks show the statistical significance (Student's unpaired *t*-test). (*) *P* < 0.05, (**) *P* < 0.01, and (***) *P* < 0.001. Not significant, *P* > 0.05. Gray crosses indicate the individual replicate values.

Next, the effect of Spt5 depletion on splicing was investigated for the same intron-containing genes (*ACT1*, *RPS13*, and *ECM33*). In order to distinguish defects at different stages of splicing catalysis, reverse transcriptase real-time quantitative PCR (RT-qPCR) assays were performed using primers that distinguish unspliced pre-mRNA, lariat (excised intron lariat or lariat-exon 2) and spliced exons (Fig. 3A). An increase in 3′SS and 5′SS or BP signals is indicative of pre-mRNA accumulation and a first step splicing defect. Increased signals for 3′SS and lariat are indicative of a second step splicing defect (lariat-exon 2). Increased lariat signal only (without 3′SS or BP accumulation) suggests accumulation of the excised intron-lariat. RT-qPCR of lariat species involves using a primer that spans the conserved branchsite of the lariat. Of the genes tested, only *ACT1* lariats can be reliably measured this way.

RT-qPCR on total (steady-state) RNA showed that depletion of Spt5 resulted in accumulation of pre-mRNA for *ACT1* (BP and 3′SS signals) *ECM33* (5′SS signal) and *RPS13* (5′SS signal), indicating a first step defect in pre-mRNA splicing (Fig. 3B; Supplemental Fig. S1). In the case of *ACT1*, we were also able to quantify lariat species, which shows that depletion of Spt5 resulted in a reduction in lariat signal, supporting a first step splicing defect (Fig. 3B; Supplemental Fig. S1). The observation that the levels of the spliced mRNAs were not significantly changed likely reflects the relatively short Spt5 depletion time as well as the relatively mild splicing defect.

### Spt5 interacts with snRNPs

**FIGURE 4.**
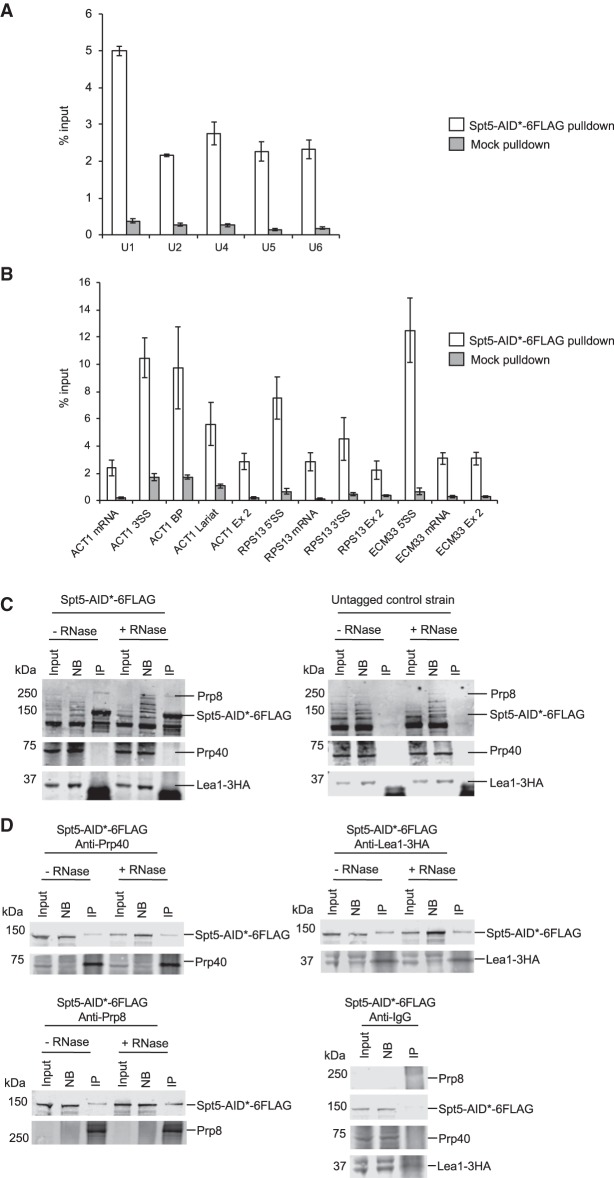

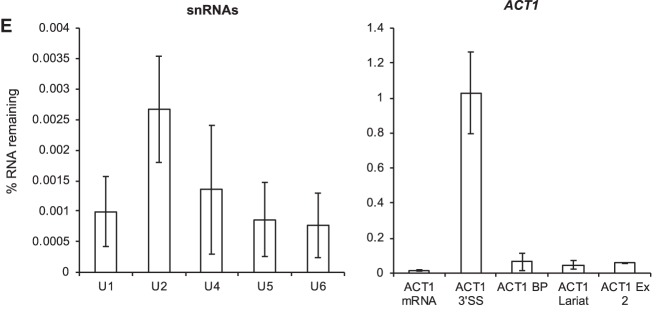
Spt5 interacts with snRNPs. (*A*) RIP experiment in which Spt5-AID*-6Flag was pulled down followed by RT-qPCR using primers against snRNAs U1, U2, U4, U5, and U6 (white bars). A mock pulldown was also performed (gray bars). Data are normalized to the input. Error bars, standard deviation of three biological replicates. (*B*) RIP experiment in which Spt5-AID*-6Flag was pulled down followed by RT-qPCR using primers for the intron-containing genes *ACT1*, *RPS13*, and *ECM33* (white bars). A mock pulldown was also performed (gray bars). Data are normalized as percentage of input (% input). Error bars, standard deviation of three biological replicates. (*C*) Western blots from a coimmunoprecipitation experiment in which Spt5-AID*-6Flag was pulled down using anti-Flag antibody with or without RNase treatment, blotted and probed with anti-Prp40 (U1 snRNP), anti-Lea-3HA (U2 snRNP), and anti-Prp8 (U5 snRNP) antibodies, then probed with the anti-Flag antibody. Additionally, an untagged Spt5 control strain with Lea1-3HA tagged was used for a pulldown with the Flag antibody as a negative control. Input (10%), nonbound (NB), and immunoprecipitation (IP) samples were loaded. (*D*) Western blots from coimmunoprecipitation experiments in which the U1 snRNP, U2 snRNP, or U5 snRNP were pulled down using anti-Prp40, anti-HA (for Lea1-HA), or anti-Prp8, respectively, in the Spt5-AID*-6Flag strain with Lea1-3HA tagged. Additionally, a negative rabbit IgG control was included in which rabbit IgG was used for a pulldown with the Spt5-AID*-6Flag strain with Lea1-3HA tagged. Input (10%), nonbound, and immunoprecipitation (IP) samples were loaded. The blot was probed with antibodies against Prp8 (U5 snRNP), Flag (Spt5-AID*-6Flag), Prp40 (U1 snRNP), and HA (Lea1-3HA) (U2 snRNP). (*E*) RT-qPCR analysis to determine efficiency of RNase treatment of samples used for coimmunoprecipitation in Figure 4C,D. Primers were used against snRNAs U1, U2, U4, U5, and U6 and *ACT1* (mRNA, 3′SS, BP, Lariat, and Exon 2). Data are shown as % RNA remaining relative to conditions without RNase treatment. Mean of two biological replicates. Error bars, standard deviation.

RNA immunoprecipitation (RIP) was performed in which Spt5-AID*-6Flag was pulled down using a Flag antibody and associated RNA was purified followed by RT-qPCR to detect any association of Spt5 with U1, U2, U4, U5, and U6 spliceosomal RNAs. RIP analysis showed Spt5 interacting mostly with the U1 snRNA, and also with U2, U4, U5, and U6 snRNAs significantly above background (Fig. 4A). RT-qPCR of intron-containing transcripts showed Spt5 pulling down more pre-mRNAs in comparison with spliced RNAs, in agreement with previous studies, which found that Spt5 exhibited intron bias and interaction with nascent pre-mRNAs (especially introns) (Fig. 4B; Moore et al. 2006; Battaglia et al. 2017). To investigate the possibility of an interaction between Spt5 and spliceosomal proteins, coimmunoprecipitation experiments were performed in which Spt5-AID*-6Flag was pulled down using a Flag antibody, followed by western blotting with antibodies against Prp40 (U1), Lea1-3HA (U2), and Prp8 (U5). As shown in Figure 4C, Prp8 was specifically coimmunoprecipitated with Spt5-AID*-6Flag and, as the addition of RNase did not affect the coimmunoprecipitation, this interaction appears to be RNA-independent. No pulldown of Prp8 was detected using a control strain with untagged Spt5, confirming the specificity of the coimmunoprecipitation. Although Prp40 (U1 snRNP) and Lea1 were not detected in the pulldown of Spt5 (Fig. 4C), immunoprecipitation of Prp40, Lea1, and Prp8 each coimmunoprecipitated Spt5 in an RNase-resistant manner (Fig. 4D). Therefore, Spt5 appears to interact with several core spliceosomal proteins, but only the coimmunoprecipitation between Spt5-AID*-6Flag and Prp8 was reciprocal. RT-qPCR analysis demonstrated the effectiveness of the RNase treatment for both snRNAs and pre-mRNA (Fig. 4E).

### The effect of Spt5 depletion on cotranscriptional recruitment of the U5 snRNP is not Paf1-dependent

**FIGURE 5.**
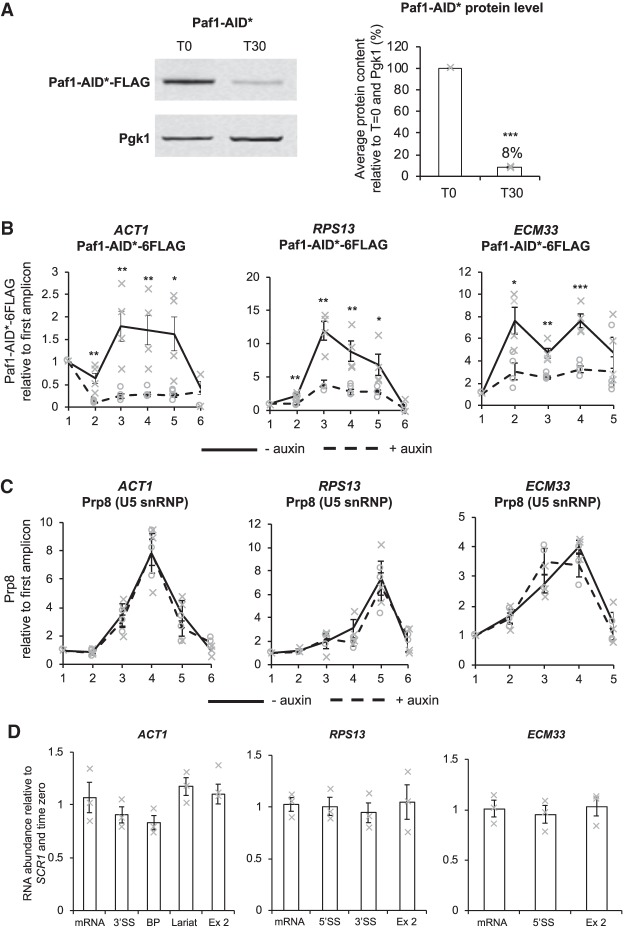
Paf1 depletion does not affect recruitment of U5 snRNP or pre-mRNA splicing. (*A*) Western blot probed with anti-Flag and anti-Pgk1 as a loading control. T0, samples taken before or T30, 30 min after addition of auxin. Paf1-AID* depletion was quantified and shown as the percentage mean of three biological replicates of T30 relative to T0 values and normalized to the Pgk1 signal. Error bars, standard error of the mean. Gray crosses indicate the individual replicate values. (*B*) Anti-Flag (Paf1-AID*) and (*C*) anti-Prp8 (U5 snRNP) ChIP followed by qPCR analysis of the intron-containing genes: *ACT1*, *RPS13*, *ECM33,* 0 min (no auxin; solid black line) and 30 min (+auxin; dashed black line) after auxin addition to depleting Paf1-AID*. *X*-axes show amplicons used for ChIP-qPCR analysis (see Fig. 1B). Data are presented as mean percentage of input relative to the first amplicon of each gene. Mean of at least three biological replicates. Error bars, standard error of the mean. Asterisks show the statistical significance (Student's unpaired *t*-test). (*) *P* < 0.05, (**) *P* < 0.01, and (***) *P* < 0.001. Not significant, *P* > 0.05. Gray crosses indicate the individual replicate values without auxin and β-estradiol and gray circles indicate the individual replicate values 40 min after auxin and β-estradiol addition. (*D*) RT-qPCR analysis of total RNA from the intron-containing genes *ACT1*, *RPS13*, and *ECM33* after 30 min of depletion of Paf1-AID*. (*A*) Normalized to the *SCR1* RNAPIII transcript and time zero (no auxin). Primers used detected pre-mRNA (5′SS or BP and 3′SS), lariat (excised intron or intron–exon 2), exon 2 (ex 2), and mRNA (see Fig. 3A for cartoon). Mean of three biological replicates. Error bars, standard error of the mean. Gray crosses indicate the individual replicate values.

To test whether the effect of Spt5 depletion on cotranscriptional spliceosome assembly was due to loss of Paf1C, a core member of the complex, Paf1, was depleted by the AID system and effects on cotranscriptional spliceosome assembly were determined. Western blotting showed that 30 min of auxin treatment resulted in a significant reduction in Paf1-AID* to, on average, 8% relative to cells without auxin treatment (Fig. 5A). The ChIP-qPCR analysis showed that, in addition to being depleted in whole cell extracts, Paf1 was significantly depleted across the intron-containing genes tested after auxin treatment (Fig. 5B). However, in contrast to the effect of depleting Spt5, ChIP-qPCR analysis showed no significant change in the occupancy of the U5 snRNP at *ACT1, ECM33* or *RPS13*, following 30 min of Paf1-AID* depletion, relative to conditions prior to auxin addition (Fig. 5C; Supplemental Fig. S2). Nor were consistent changes in U1 or U2 snRNP occupancy observed (data not shown).

To determine whether Paf1-AID* depletion affected pre-mRNA splicing, RT-qPCR was performed, as described above, on total (steady-state) RNA. No significant change in the levels of the pre-mRNA, spliced exons or exon 2 was observed for *ACT1*, *RPS13*, and *ECM33* following depletion of Paf1-AID*, relative to conditions prior to auxin addition (Fig. 5D).

## DISCUSSION

There is some evidence that core members of the transcription elongation complex interact with splicing factors (Brés et al. 2005; Moore et al. 2006; Cao et al. 2015; Li et al. 2016), and can affect splicing outcome (Lindstrom et al. 2003; Burckin et al. 2005; Xiao et al. 2005a; Diamant et al. 2012; Liu et al. 2012; Shetty et al. 2017). However, there is currently little insight into how the core transcription elongation machinery affects cotranscriptional splicing or whether observed effects are direct or indirect (for review, see Neugebauer 2002; Merkhofer et al. 2014). Here, using the AID system to conditionally deplete transcription elongation factor Spt5, we provide insight into the contribution of Spt5 to pre-mRNA splicing in *S. cerevisiae*.

**FIGURE 6.**
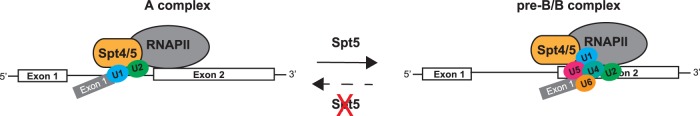
Model: a role for Spt5 in cotranscriptional spliceosome assembly. In wild-type conditions (without Spt5 depletion), Spt5 facilitates cotranscriptional spliceosome assembly by promoting stable recruitment of the U5 snRNP. This may be mediated, either directly or indirectly, by the interaction between Spt5 and core members of the spliceosome, although it is unclear whether the interaction occurs already at the prespliceosome stage. Upon Spt5 depletion (indicated by the red cross), the U5 snRNP is either not recruited or does not remain stably associated, so that pre-B/B complexes or later complexes do not form, or form and then rapidly disassemble, leading to defects in splicing catalysis.

ChIP-qPCR, using antibodies against individual snRNP components, is a well-established method to monitor stepwise cotranscriptional spliceosome assembly (Kotovic et al. 2003; Görnemann et al. 2005; Lacadie and Rosbash 2005; Tardiff and Rosbash 2006). In particular, Prp8 is a reliable indicator of the presence of the U5 snRNP, as the absence of Prp8 results in failure to form stable U5 snRNP or U4/U6.U5 tri-snRNP or their failure to assemble into spliceosomes (Brown and Beggs 1992). It was also shown previously that prespliceosomes can form in vivo in the absence of the U5 snRNP (Tardiff and Rosbash 2006). Following Spt5 depletion, we observed normal cotranscriptional recruitment of the U1 and U2 snRNPs but not of U5 snRNP to intron-containing genes (Fig. 2), indicating unperturbed cotranscriptional assembly of the prespliceosome (A complex) but possible failure to form pre-B complex. However, the observation of a low level signal for U5 snRNP may indicate that transient pre-B complex forms but, in the absence of Spt5, dissociates, without conversion to B complex (Figs. 2C, 6). Single molecule imaging analyses of spliceosome assembly in vitro have shown that individual stages of stepwise spliceosome assembly, including tri-snRNP association with the prespliceosome, are reversible in *S. cerevisiae* (Hoskins et al. 2011), and there is separate evidence that both steps of splicing can be reversed in vitro (Tseng and Cheng 2008). Therefore, we cannot rule out the possibility that stable B complex forms and is rapidly converted to activated spliceosome that is itself unstable and is rapidly disassembled.

Although Spt5 promotes transcription elongation, the effects of Spt5 depletion on U5 snRNP recruitment are not simply due to altered transcription. Under the Spt5 depletion conditions used, the transcript levels did not significantly change (exon 2 in Fig. 3B), nor did RNAPII occupancy (Fig. 2D) change for the intron-containing genes tested. Moreover, changes to transcription would be predicted to affect the cotranscriptional recruitment of U1, U2, and U5 snRNPs similarly, whereas U1 and U2 snRNP recruitment was not changed by Spt5 depletion.

A defect in the cotranscriptional formation of spliceosomes can explain the observed mild splicing defect (Fig. 3). This is consistent with previous studies in which mutations in Spt5 caused splicing defects in *S. cerevisiae*, and where depletion of Spt5 resulted in pre-mRNA accumulation in *S. pombe* (Lindstrom et al. 2003; Burckin et al. 2005; Shetty et al. 2017). It has been demonstrated that splicing is more efficient when cotranscriptional (Aslanzadeh et al. 2018), so that, although Spt5 likely does not affect post-transcriptional splicing, this does not compensate for lack of cotranscriptional splicing, explaining the relatively modest splicing defect observed when Spt5 was depleted.

How might this effect of Spt5 on cotranscriptional spliceosome assembly be mediated? Coimmunoprecipitation experiments showed a reciprocal association of Spt5 and Prp8 (Fig. 4C,D). We also observed Prp40 (U1 snRNP) pulldown of Spt5, which is in agreement with a previous study (Moore et al. 2006). However, Prp40 (U1) and Lea1 (U2) coimmunoprecipitated Spt5 in a nonreciprocal manner, which might suggest that these interactions occur in the context of the spliceosome. Indeed, RIP experiments showed that Spt5 interacted with all five spliceosomal snRNAs (Fig. 4A), with U1 snRNA being pulled down the most, and that the intronic regions of the pre-mRNAs were enriched in the pulldowns compared with the exons (Fig. 4B). Although the interactions of Spt5 with the snRNP proteins are reproducibly all resistant to RNase treatment (Fig. 4C,D), the intronic regions of *ACT1* (the only transcript analyzed by RT-qPCR after RNase treatment) were relatively protected against the RNase treatment compared with the mRNA splice junction and the snRNAs, therefore it cannot be ruled out that the Spt5 interactions with splicing factors are intron-mediated. While our data are consistent with direct interactions between Spt5 and splicing factors occurring in vivo, we cannot exclude the possibility that interactions may be indirect or form post-lysis (Mili and Steitz 2004). Assuming that these interactions occur in vivo, as Spt5 is a transcription elongation factor, they presumably occur at sites of transcription elongation.

The amino-terminal region of Prp8 (U5 snRNP) has been reported to interact with several U1 snRNP proteins, including Prp40 (for review, see Grainger and Beggs 2005). Interestingly, the conserved WW domains of Prp40 were proposed to bind the amino-terminal part of Prp8p in yeast, through proline-rich motifs (Abovich and Rosbash 1997; Wiesner et al. 2002), possibly bridging interactions across the intron. In a functional analysis of the role of the Prp40 WW domains in splicing, Görnemann et al. (2011) found that deletion of the Prp40 WW domains reduced cotranscriptional U5 snRNP recruitment without affecting U1 or U2 snRNP recruitment, similar to our findings for Spt5 depletion. It is therefore tempting to speculate that Spt5 may promote interaction between Prp8, in the U5 snRNP, and Prp40 (and potentially other U1 snRNP proteins) during tri-snRNP recruitment, thereby facilitating stable B complex formation cotranscriptionally, as indicated in our proposed model (Fig. 6).

Together, these data provide insight into how Spt5 could affect pre-mRNA splicing, by modulating cotranscriptional recruitment or stable association of the U5 snRNP and/or tri-snRNP during spliceosome assembly, most likely by direct or indirect interaction with the spliceosome (Fig. 6). We further show that the defect caused by Spt5 depletion is apparently not a consequence of failure to recruit the Paf1 complex to RNAPII (Fig. 5), more directly implicating Spt5 itself, rather than recruitment of downstream transcription factors.

These results provide evidence of a role for a transcription elongation factor in cotranscriptional spliceosome assembly and thereby for the recruitment model of cotranscriptional splicing. As Spt5 is highly conserved, it will be of interest to determine whether Spt5 plays a similar role in cotranscriptional spliceosome assembly in higher eukaryotes, which is crucial for cotranscriptional regulation of alternative splicing.

## MATERIALS AND METHODS

### Yeast strains and growth conditions

Yeast strains are listed in Table 1. Spt5 was carboxy-terminally AID*-6Flag tagged in a W303 strain containing a centromeric plasmid that allowed conditional induction of OsTIR1 using the β-estradiol system (McIsaac et al. 2014; Mendoza-Ochoa et al. 2018). Paf1 was carboxy-terminally AID*-6Flag tagged in the YBRT background strain. For tagging, a plasmid was used with the AID* cassette comprised of the AUX/IAA (AID*) recognition motif for auxin-mediated depletion and a 6× Flag tag for immunodetection (Morawska and Ulrich 2013).

**TABLE 1.**
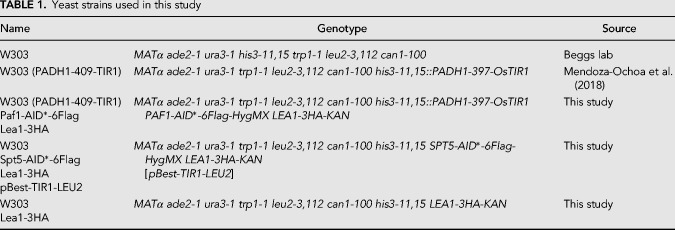
Yeast strains used in this study

### Auxin time course experiments

To induce TIR1 using the β-estradiol system, cells, grown in leucine-deficient yeast minimal media (YMM) to OD_600_ 0.7, were treated with 10 µM β-estradiol (Sigma-Aldrich #E8875; dissolved in 100% ethanol) to induce TIR1 expression and 0.75 mM Indole-3-acetic acid (IAA; auxin) (Acros organics #122160100) to deplete Spt5-AID*, for 40 min. To deplete Paf1-AID*, cells grown in YPDA medium to OD_600_ 0.7 were treated with 0.75 mM IAA for 30 min. After incubation with auxin, samples were taken for protein, RNA and chromatin extraction as described below.

### Protein sample preparation and western blotting

Protein samples were prepared using a NaOH lysis and trichloroacetic acid (TCA) precipitation protocol (Volland et al. 1994). For western blotting, 25 µg of protein was run on a NuPAGE 4%–12% Bis-Tris gel (Invitrogen #NP0323BOX) at 180 V in 1× MOPS-SDS buffer (Invitrogen #1862491). Proteins were transferred to a Bio-Rad nitrocellulose membrane (0.2 µm, #LC2009) using a semi-wet transfer unit (Bio-Rad) at 100 V for 1 h at 4°C in Tris-Glycine transfer buffer (200 mM Tris, 1.5 M glycine) with 10% methanol. After transfer, proteins of interest were visualized using the Odyssey infrared imaging system (LI-COR Bioscience), and quantified by the median method of the Odyssey software. Data were normalized against the 3-Phosphoglycerate Kinase (Pgk1) loading control. Primary and secondary antibodies used are listed in Table 2.

**TABLE 2.**
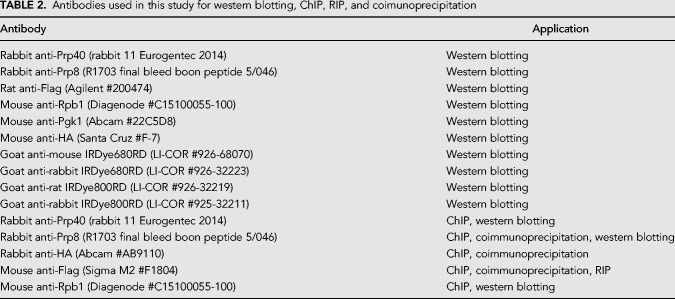
Antibodies used in this study for western blotting, ChIP, RIP, and coimunoprecipitation

### RNA preparation and RT-qPCR

RNA was extracted using a modified GTC:phenol method and RT-qPCR was performed as described in Alexander et al. (2010b). A list of primers used for RT-qPCR can be provided upon request.

### Chromatin immunoprecipitation (ChIP)

Fifty milliliters of culture at OD__600__ 0.8 were cross-linked in 1% (w/v) formaldehyde for 10 min with shaking at room temperature. The reaction was stopped by incubating the cells for 5 min with 2.5 mL of 2.5 M glycine. Cells were harvested by centrifugation and washed twice in ice-cold 1× PBS. Cell pellets were resuspended in 350 µL FA1 buffer (50 mM HEPES-KOH pH 7.5, 140 mM NaCl, 1 mM EDTA pH 8.0, 1% Triton X-100, 0.1% sodium deoxycholate, one complete EDTA-free proteinase inhibitor tablet [Roche #11836145001], PhosSTOP tablets [Sigma Aldrich #000000004906845001]) and 350 µL zirconia beads. The cells were disrupted using the Mini-Beadbeater-24 (BioSpec Products) twice at 2000 rpm for 2 min with 2 min on ice in between. The sample was separated from the beads by centrifugation at 1000*g* for 2 min. The sample was centrifuged at 20,000*g* for 15 min at 4°C. The pellet was resuspended in 600 µL FA1 buffer, and the sample sonicated using a New Twin Biorupt sonicator (Diagenode) for 15 cycles 30 sec on and 30 sec off. The sample was centrifuged at 20,000*g* for 30 min at 4°C and the supernatant containing solubilized chromatin was retained. For immunoprecipitation, the appropriate amount of chromatin was incubated in 20 µL Protein A/G Dynabeads (Life Technologies #10001D/10003D) conjugated to the antibody on a rotating wheel overnight at 4°C. A list of the antibodies for ChIP can be found in Table 2.

The beads were washed three times in FA1 buffer, twice in FA2 buffer (50 mM HEPES-KOH pH 7.5, 0.5 M NaCl, 1 mM EDTA pH 8.0, 1% Triton X-100, 0.1% sodium deoxycholate), twice in FA3 buffer (10 mM Tris-HCl pH 8.0, 250 mM LiCl, 1 mM EDTA pH 8.0, 0.5% NP-40, 0.5% Na deoxycholate) and once in Tris-EDTA pH 8.0 0.05% TWEEN-20. Crosslinking was reversed with 150 µL elution buffer (50 mM Tris-HCl, 10 mM EDTA, 1% SDS) and 3 µL Proteinase K (25 mg/mL) and incubated at 42°C for 2 h and 65°C overnight, shaking. An input sample equal to 10% of the protein that was used for the immunoprecipitation was prepared and crosslinking was reversed as above. The QIAGEN mini column clean-up kit was used to purify DNA according to the manufacturer's instructions, and DNA eluted in 400 µL of 10 mM Tris pH 8.0. Samples were analyzed by qPCR as described above using primers that can be provided upon request. The ChIP data were normalized using the relative threshold cycle (*C*_t_) values for each sample. ChIP data are presented as percentage of input normalized to the first amplicon of each gene.

### Coimmunoprecipitation

Two hundred and fifty milliliters of culture at OD_600_ 0.8 was harvested by centrifugation and washed twice in ice-cold 1× PBS. The cell pellet was resuspended in 900 µL lysis buffer (50 mM Tris-HCl pH 7.5, 2 mM Mg_2_Cl_2_, 150 mM NaCl, 0.2% NP-40, one complete EDTA-free proteinase inhibitor tablet [Roche #11836145001]) and 400 µL zirconia beads. Cells were lysed using a Mini-Beadbeater-24 as described above. The sample was centrifuged at 1000*g* for 2 min, the supernatant collected and further centrifuged at 20,000*g* for 30 min at 4°C and used for immunoprecipitation. Fifty microliters Protein A/G Dynabeads (Life Technologies #10001D/10003D) conjugated to antibody were incubated with 1 mg of protein on a rotating wheel for 1 h at room temperature. The beads were washed eight times in lysis buffer. Twenty microliters of loading buffer was added to the beads, input and nonbound samples, which were boiled for 10 min before loading on a 4%–12% Bis-Tris gel followed by western blotting as described above. A list of antibodies used for coimmunoprecipitation and subsequent western blotting can be found in Table 2.

For RNase treatment, 1 mg of protein was incubated with 100 µg/mL RNase A (Sigma Aldrich #R4642) for 30 min at room temperature prior to immunoprecipitation. The efficiency of RNase treatment was verified by RNA extraction and RT-qPCR as described above.

### RNA immunoprecipitation

RNA immunoprecipitation was performed using a protocol modified from Churchman and Weissman (2012). Cells at OD_600_ 0.8 were harvested by centrifugation and subjected to cryogeniclysis and DNase treatment as described in Churchman and Weissman (2012). For immunoprecipitation, 1 mg of lysate was incubated with 20 µL of dynabeads Protein A/G Dynabeads (Life Technologies #10001D/10003D) conjugated to the antibody on a rotating wheel for 2 h at 4°C. As a negative control, a mock pulldown using IgG was performed. The Flag antibody used for immunoprecipitation can be found in Table 2. The beads were washed four times in lysis buffer. RNA was extracted and purified from input and pulldown samples using the Qiagen miRNeasy mini kit according to the manufacturer's instructions. RIP data are presented as percentage of input. A list of primers used for RT-qPCR to detect snRNAs can be provided upon request.

## SUPPLEMENTAL MATERIAL

Supplemental material is available for this article.

## Supplementary Material

Supplemental Material
