# Supplementary material for: Spt5 modulates cotranscriptional spliceosome assembly in Saccharomyces cerevisiae
Source: RNA. 2019 Oct;25(10):1298–310. doi: 10.1261/rna.070425.119 (PMC6800482; doi:10.1261/rna.070425.119)
Supplement: Supplemental Material [file supp_25_10_1298__index.html]

Spt5 modulates cotranscriptional spliceosome assembly in Saccharomyces cerevisiae — Supplemental Material 

# Spt5 modulates cotranscriptional spliceosome assembly in *Saccharomyces cerevisiae*

## Supplemental Material

- Supplemental\_Figure\_Legends.docx
- Supplemental\_Figure\_S1.pdf
- Supplemental\_Figure\_S2.pdf
