# Supplementary material for: Spt5 modulates cotranscriptional spliceosome assembly in Saccharomyces cerevisiae
Source: RNA. 2019 Oct;25(10):1298–310. doi: 10.1261/rna.070425.119 (PMC6800482; doi:10.1261/rna.070425.119)
Supplement: Supplemental Material [file supp_070425.119_Supplemental_Figure_Legends.docx]

**Supplementary Figure S1.**

RT-qPCR analysis of the intron-containing transcripts of *ACT1* (A), *RPS13* (B) and *ECM33* (C) before (T0) and after (T40) depletion of Spt5-AID*, normalised to the *SCR1* RNAPIII transcript. Mean of 3 biological replicates. Error bars = standard error of the mean. Gray crosses indicate the individual replicate values. Due to different efficiencies of PCR probes, the levels of different RNA species cannot be closely compared.

**Supplementary Figure S2.**

RT-qPCR analysis of the intron-containing transcripts of *ACT1* (A), *RPS13* (B) and *ECM33* (C) before (T0) and after (T30) depletion of Paf1-AID*, normalised to the *SCR1* RNAPIII transcript. Mean of 3 biological replicates. Error bars = standard error of the mean. Gray crosses indicate the individual replicate values. Due to different efficiencies of PCR probes, the levels of different RNA species cannot be closely compared.
