# Supplementary figures and images for: Spt5 modulates cotranscriptional spliceosome assembly in Saccharomyces cerevisiae
Source: RNA. 2019 Oct;25(10):1298–310. doi: 10.1261/rna.070425.119 (PMC6800482; doi:10.1261/rna.070425.119)

**Supplementary Figure S1 (related to Figure 3C)**

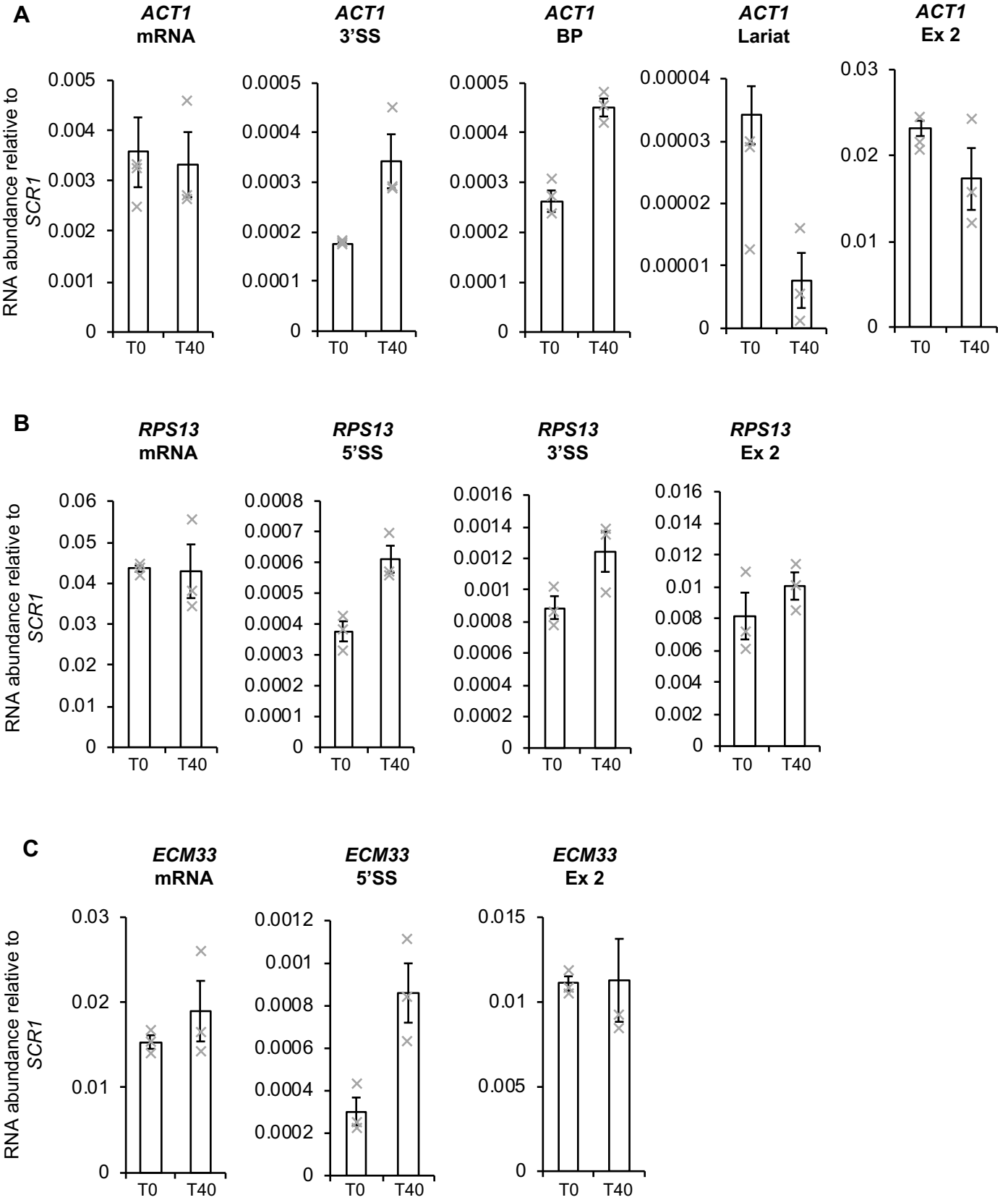

Supplement: Supplemental Material [file supp_070425.119_Supplemental_Figure_S1.pdf]

Supplementary Figure S2 (related to Figure 5D)

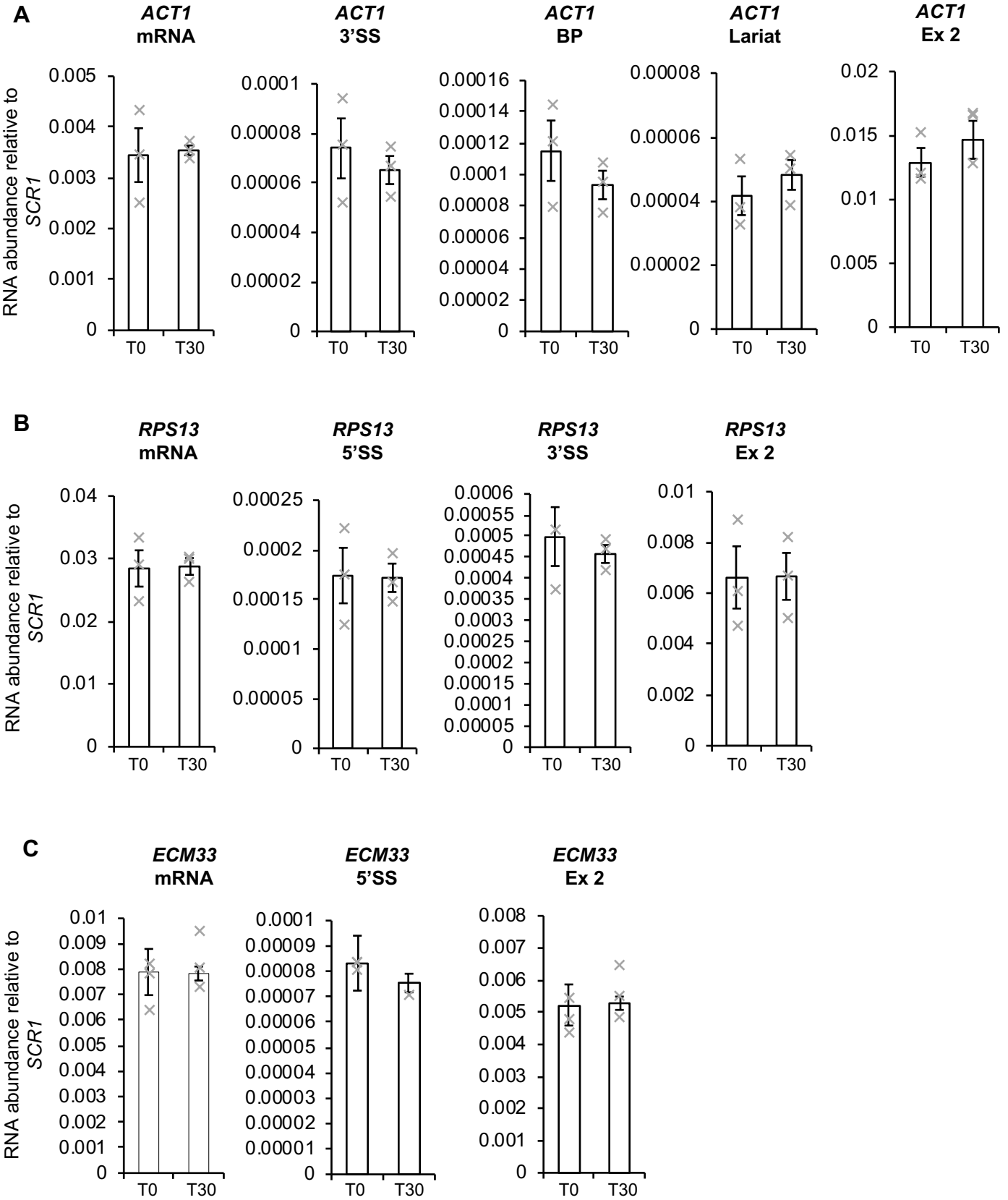

Supplement: Supplemental Material [file supp_070425.119_Supplemental_Figure_S2.pdf]
